# Poly[[hexa-μ-aqua-diaqua­bis­(μ_4_-dihydrogen benzene-1,2,4,5-tetra­carboxyl­ato)magnesiumdisodium] dihydrate]

**DOI:** 10.1107/S1600536812024634

**Published:** 2012-06-13

**Authors:** Dan Zhao, Peng Liang, Yan-Feng Li, Sen Qiu, Jun-Ran Ren

**Affiliations:** a Department of Physics and Chemistry, Henan Polytechnic University, Jiaozuo, Henan 454000, People’s Republic of China

## Abstract

The asymmetric unit of the title compound, {[MgNa_2_(C_10_H_4_O_8_)_2_(H_2_O)_8_]·2H_2_O}_*n*_, contains one octa­hedrally coordin­ated Mg^II^ atom (site symmetry 2/*m*), one octahedrally coordinated Na^I^ atom (site symmetry 2) and one half of the dihydrogen benzene-1,2,4,5-tetra­carboxyl­ate (btec) ligand, the second half of the ligand being generated by a twofold rotation axis. The basic framework of the title compound features infinite (–Na–Na–Mg–)_*n*_ chains along [10-1] with the metal cations bridged by the coordinating water molecules. The chains are isolated from each other by μ_4_-bridging btec ligands, which form inter­molecular O—H⋯O hydrogen bonds to uncoordinated water mol­ecules and the coordinated water mol­ecules of a neighbouring chain. In each btec ligand, there are also intramolecular O—H⋯O hydrogen bonds.

## Related literature

For structures based on the H_4_btec ligand, see: Gong & Zhang (2011 ▶); Liu *et al.* (2009 ▶, 2010 ▶); Zhang *et al.* (2007 ▶).
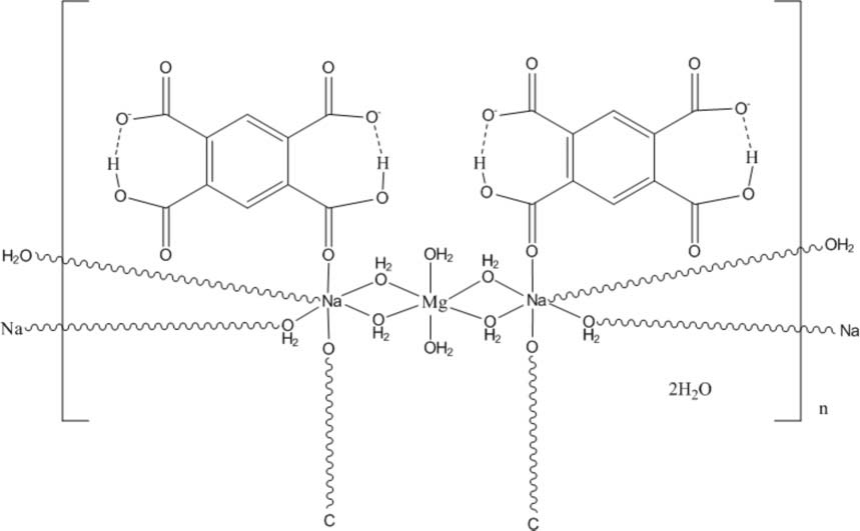


## Experimental

### 

#### Crystal data

[MgNa_2_(C_10_H_4_O_8_)_2_(H_2_O)_8_]·2H_2_O
*M* *_r_* = 754.71
Monoclinic, 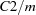

*a* = 7.3335 (13) Å
*b* = 20.155 (4) Å
*c* = 10.4450 (18) Å
β = 103.325 (3)°
*V* = 1502.3 (5) Å^3^
*Z* = 2
Mo *K*α radiation
μ = 0.20 mm^−1^
*T* = 296 K
0.20 × 0.05 × 0.05 mm

#### Data collection

Bruker APEXII CCD area-detector diffractometer
Absorption correction: multi-scan (*SADABS*; Sheldrick, 1996 ▶) *T*_min_ = 0.961, *T*_max_ = 0.990
4088 measured reflections
1440 independent reflections
1272 reflections with *I* > 2σ(*I*)
*R*_int_ = 0.022

#### Refinement

*R*[*F* ^2^ > 2σ(*F* ^2^)] = 0.030
*wR*(*F* ^2^) = 0.089
*S* = 1.08
1440 reflections
122 parameters
H-atom parameters constrained
Δρ_max_ = 0.23 e Å^−3^
Δρ_min_ = −0.21 e Å^−3^

### 

Data collection: *APEX2* (Bruker, 2008 ▶); cell refinement: *SAINT* (Bruker, 2008 ▶); data reduction: *SAINT*; program(s) used to solve structure: *SHELXS97* (Sheldrick, 2008 ▶); program(s) used to refine structure: *SHELXL97* (Sheldrick, 2008 ▶); molecular graphics: *SHELXTL* (Sheldrick, 2008 ▶); software used to prepare material for publication: *SHELXTL*.

## Supplementary Material

Crystal structure: contains datablock(s) I, global. DOI: 10.1107/S1600536812024634/ez2296sup1.cif

Structure factors: contains datablock(s) I. DOI: 10.1107/S1600536812024634/ez2296Isup2.hkl

Additional supplementary materials:  crystallographic information; 3D view; checkCIF report

## Figures and Tables

**Table 1:** Hydrogen-bond geometry (Å, °)

*D*—H⋯*A*	*D*—H	H⋯*A*	*D*⋯*A*	*D*—H⋯*A*
O3—H3⋯O2	1.06	1.35	2.3827 (17)	163
O5—H5⋯O7	0.90	1.83	2.7313 (14)	173
O6—H6⋯O4^i^	0.90	1.97	2.8519 (15)	167
O7—H7⋯O2^ii^	0.86	1.91	2.7699 (14)	173
O8—H8⋯O3^iii^	0.85	1.94	2.7779 (14)	172
O9—H9⋯O4^ii^	0.85	1.91	2.7559 (14)	171

Symmetry codes: (i) 
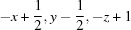
; (ii) 
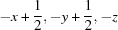
; (iii) 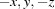.

**Fig. 1.**
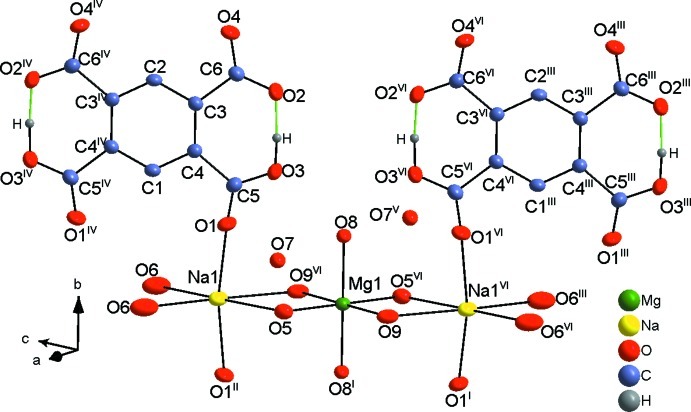
A view of the title compound showing the atom-numbering scheme. Displacement ellipsoids are drawn at the 50% probability level and hydrogen atoms are omitted for clarity, except for intramolecular hydrogen bonding H atoms in the btec ligands (indicated as pea green lines). [Symmetry codes: (i) -x, -y, -z; (ii) x, -y, z; (iii) x - 1, y, z - 1; (iv) -x + 1, y, -z + 1; (v) x - 1, y, z; (vi) -x, y, -z].

**Fig. 2.**
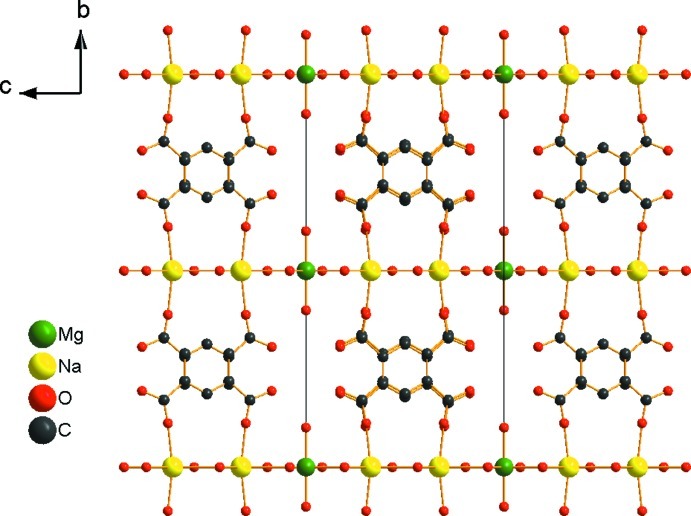
View of the crystal structure of the title compound along the a-axis.

## supplementary crystallographic information

### Comment

In recent years, much attention has been paid to coordination polymer materials based on covalent interactions or supramolecular contacts, and huge numbers of novel compounds with interesting structures and topologies have been reported. As part of this research benzene-1,2,4,5-tetracarboxylate (btec) can be used as a ligand to form various supramolecular architectures with its four rigid carboxyl groups (Gong *et al.*, 2011; Liu *et al.*, 2009; Liu *et al.*, 2010; Zhang *et al.*, 2007). In order to enrich this family of compounds, we used the hydrothermal method to synthesise the title compound, a new sodium(I)-magnesium(II) complex, that is, Na_2_Mg(btec)_2_(H_2_O)_8_.2(H_2_O), where btec = benzene-1,2,4,5-tetracarboxylate, and we determined its structure by single-crystal X-ray diffraction.

As shown in Fig. 1, the asymmetric unit of the title compound contains one octahedrally coordinated magnesium atom, one octahedrally coordinated sodium atom and half a benzene-1,2,4,5-tetracarboxylate (btec) ligand. Each btec ligands contains two intramolecular O–H···O hydrogen bonds, with the H atoms bonded to atoms O3 and O2, and connects two Na atoms in a *µ*_2_– manner. Each Na atom is coordinated by two *cis* carboxylate oxygen atoms from two btec ligands and by four water molecules, while each Mg is coordinated by six water molecules. The Na–O bond distances range from 2.2669 (12) to 2.6146 (18) Å, while the Mg–O bond lengths are slightly longer ranging from 2.0301 (14) to 2.1008 (14) Å. Furthermore, NaO_6_ octahedra and MgO_6_ octahedra are connected *via* coordinated water molecules to form a one-dimensional infinite (–Na–Na–Mg–)_n_ chain, as shown in Fig. 2. O—H···O hydrogen bonds link the coordinated and uncoordinated water molecules to neighbouring btec ligands (Table 1).

### Experimental

A mixture of 1,2,4,5-benzene-tetracarboxylic (0.2 g), Na_2_CO3 (0.1 g), MgO(0.05 g) and H_2_O (15 ml) was heated at 448 K for 7 d in a sealed 25 ml Teflon-lined stainless steel vessel under autogenous pressure. After cooling to room temperature at a rate of 5 C h^-1^, colourless prismatic crystals were obtained in low yield.

### Refinement

The H atoms of C atoms were positioned geometrically and refined with a riding model, with C—H = 0.93 Å and *U*_iso_(H) = 1.2U_eq_(C). The water H atoms were located in difference Fourier maps, and then refined with a riding model, with *U*_iso_(H) = 1.5U_eq_(O).

### Crystal data

**Table:**

[MgNa_2_(C_10_H_4_O_8_)_2_(H_2_O)_8_]·2H_2_O	*F*(000) = 780
*M**_r_* = 754.71	*D*_x_ = 1.668 Mg m^−^^3^
Monoclinic, *C*2/*m*	Mo *K*α radiation, λ = 0.71073 Å
Hall symbol: -C 2y	Cell parameters from 2031 reflections
*a* = 7.3335 (13) Å	θ = 2.9–27.8°
*b* = 20.155 (4) Å	µ = 0.20 mm^−^^1^
*c* = 10.4450 (18) Å	*T* = 296 K
β = 103.325 (3)°	Prism, colourless
*V* = 1502.3 (5) Å^3^	0.20 × 0.05 × 0.05 mm
*Z* = 2	

### Data collection

**Table:**

Bruker APEXII CCD area-detector diffractometer	1440 independent reflections
Radiation source: fine-focus sealed tube	1272 reflections with *I* > 2σ(*I*)
Graphite monochromator	*R*_int_ = 0.022
Detector resolution: 83.33 pixels mm^-1^	θ_max_ = 25.5°, θ_min_ = 2.0°
ω scans	*h* = −8→8
Absorption correction: multi-scan (*SADABS*; Sheldrick, 1996)	*k* = −24→22
*T*_min_ = 0.961, *T*_max_ = 0.990	*l* = −11→12
4088 measured reflections	

### Refinement

**Table:**

Refinement on *F*^2^	Secondary atom site location: difference Fourier map
Least-squares matrix: full	Hydrogen site location: inferred from neighbouring sites
*R*[*F*^2^ > 2σ(*F*^2^)] = 0.030	H-atom parameters constrained
*wR*(*F*^2^) = 0.089	*w* = 1/[σ^2^(*F*_o_^2^) + (0.0445*P*)^2^ + 0.7471*P*] where *P* = (*F*_o_^2^ + 2*F*_c_^2^)/3
*S* = 1.08	(Δ/σ)_max_ < 0.001
1440 reflections	Δρ_max_ = 0.23 e Å^−^^3^
122 parameters	Δρ_min_ = −0.21 e Å^−^^3^
0 restraints	Extinction correction: *SHELXL97* (Sheldrick, 2008), Fc^*^=kFc[1+0.001xFc^2^λ^3^/sin(2θ)]^-1/4^
Primary atom site location: structure-invariant direct methods	Extinction coefficient: 0.0033 (7)

### Special details

**Table:**

Geometry. All e.s.d.'s (except the e.s.d. in the dihedral angle between two l.s. planes) are estimated using the full covariance matrix. The cell e.s.d.'s are taken into account individually in the estimation of e.s.d.'s in distances, angles and torsion angles; correlations between e.s.d.'s in cell parameters are only used when they are defined by crystal symmetry. An approximate (isotropic) treatment of cell e.s.d.'s is used for estimating e.s.d.'s involving l.s. planes.
Refinement. Refinement of *F*^2^ against ALL reflections. The weighted *R*-factor *wR* and goodness of fit *S* are based on *F*^2^, conventional *R*-factors *R* are based on *F*, with *F* set to zero for negative *F*^2^. The threshold expression of *F*^2^ > σ(*F*^2^) is used only for calculating *R*-factors(gt) *etc*. and is not relevant to the choice of reflections for refinement. *R*-factors based on *F*^2^ are statistically about twice as large as those based on *F*, and *R*- factors based on ALL data will be even larger.

### Fractional atomic coordinates and isotropic or equivalent isotropic displacement parameters (Å^2^)

**Table:**

	*x*	*y*	*z*	*U*_iso_*/*U*_eq_	
Mg1	0.0000	0.0000	0.0000	0.0220 (2)	
Na1	0.31699 (13)	0.0000	0.32732 (8)	0.0356 (3)	
O1	0.30603 (18)	0.11183 (5)	0.30357 (11)	0.0464 (3)	
C1	0.5000	0.18685 (9)	0.5000	0.0233 (4)	
H1	0.5000	0.1407	0.5000	0.028*	
O2	0.20225 (19)	0.31210 (6)	0.17351 (10)	0.0487 (4)	
C2	0.5000	0.32051 (9)	0.5000	0.0221 (4)	
H2	0.5000	0.3667	0.5000	0.027*	
O3	0.20002 (18)	0.19389 (6)	0.17286 (10)	0.0481 (4)	
H3	0.2132	0.2456	0.1607	0.072*	
C3	0.39709 (18)	0.28869 (6)	0.38857 (12)	0.0211 (3)	
O4	0.31030 (15)	0.39579 (5)	0.30165 (10)	0.0339 (3)	
C4	0.39692 (18)	0.21864 (6)	0.38884 (12)	0.0214 (3)	
O5	0.28991 (19)	0.0000	0.08188 (14)	0.0288 (3)	
H5	0.3530	0.0340	0.0564	0.043*	
C5	0.2960 (2)	0.17086 (7)	0.28270 (14)	0.0283 (3)	
O6	0.3317 (3)	0.0000	0.56419 (17)	0.0483 (5)	
H6	0.2884	−0.0368	0.5963	0.073*	
C6	0.29702 (19)	0.33570 (7)	0.28103 (13)	0.0258 (3)	
O7	0.5000	0.09605 (7)	0.0000	0.0311 (3)	
H7	0.4367	0.1223	−0.0590	0.047*	
O8	0.0000	0.10073 (7)	0.0000	0.0322 (4)	
H8	−0.0681	0.1262	−0.0560	0.048*	
O9	0.0320 (2)	0.0000	−0.19461 (14)	0.0314 (4)	
H9	0.0897	0.0325	−0.2191	0.047*	

### Atomic displacement parameters (Å^2^)

**Table:**

	*U*^11^	*U*^22^	*U*^33^	*U*^12^	*U*^13^	*U*^23^
Mg1	0.0265 (5)	0.0161 (4)	0.0222 (5)	0.000	0.0032 (4)	0.000
Na1	0.0521 (6)	0.0188 (4)	0.0328 (5)	0.000	0.0034 (4)	0.000
O1	0.0678 (8)	0.0176 (6)	0.0428 (7)	−0.0038 (5)	−0.0098 (6)	−0.0044 (5)
C1	0.0259 (10)	0.0152 (9)	0.0275 (10)	0.000	0.0037 (8)	0.000
O2	0.0697 (8)	0.0256 (6)	0.0343 (6)	0.0023 (5)	−0.0222 (6)	0.0038 (5)
C2	0.0262 (9)	0.0136 (9)	0.0258 (10)	0.000	0.0046 (8)	0.000
O3	0.0683 (8)	0.0244 (6)	0.0350 (6)	−0.0028 (5)	−0.0224 (6)	−0.0032 (5)
C3	0.0216 (7)	0.0186 (7)	0.0225 (7)	0.0007 (5)	0.0038 (5)	0.0014 (5)
O4	0.0478 (7)	0.0177 (5)	0.0335 (6)	0.0032 (4)	0.0040 (5)	0.0050 (4)
C4	0.0215 (6)	0.0185 (7)	0.0231 (7)	−0.0010 (5)	0.0031 (5)	−0.0012 (5)
O5	0.0280 (7)	0.0221 (7)	0.0363 (8)	0.000	0.0070 (6)	0.000
C5	0.0318 (8)	0.0214 (7)	0.0282 (7)	−0.0012 (6)	0.0000 (6)	−0.0036 (6)
O6	0.0713 (12)	0.0291 (8)	0.0533 (11)	0.000	0.0321 (9)	0.000
C6	0.0282 (7)	0.0211 (7)	0.0263 (7)	0.0011 (5)	0.0028 (6)	0.0028 (6)
O7	0.0357 (8)	0.0214 (7)	0.0304 (8)	0.000	−0.0046 (6)	0.000
O8	0.0419 (9)	0.0162 (7)	0.0299 (8)	0.000	−0.0094 (6)	0.000
O9	0.0469 (9)	0.0185 (7)	0.0324 (8)	0.000	0.0166 (7)	0.000

### Geometric parameters (Å, º)

**Table:**

Mg1—O8	2.0301 (14)	O2—C6	1.2691 (17)
Mg1—O8^i^	2.0302 (14)	O2—H3	1.3505
Mg1—O9	2.0992 (14)	C2—C3	1.3895 (15)
Mg1—O9^i^	2.0992 (14)	C2—C3^iv^	1.3895 (15)
Mg1—O5^i^	2.1008 (14)	C2—H2	0.9300
Mg1—O5	2.1008 (14)	O3—C5	1.2865 (17)
Mg1—Na1^i^	3.6645 (10)	O3—H3	1.0579
Mg1—Na1	3.6646 (10)	C3—C4	1.4119 (18)
Na1—O1	2.2669 (12)	C3—C6	1.5225 (18)
Na1—O1^ii^	2.2669 (12)	O4—C6	1.2301 (17)
Na1—O6	2.4515 (19)	C4—C5	1.5250 (18)
Na1—O5	2.5252 (16)	O5—H5	0.9007
Na1—O6^iii^	2.564 (2)	O6—Na1^iii^	2.564 (2)
Na1—O9^i^	2.6146 (18)	O6—H6	0.9011
Na1—Na1^iii^	3.9692 (17)	O7—H7	0.8628
O1—C5	1.2088 (18)	O8—H8	0.8481
C1—C4	1.3876 (15)	O9—Na1^i^	2.6146 (17)
C1—C4^iv^	1.3876 (15)	O9—H9	0.8510
C1—H1	0.9300		
			
O8—Mg1—O8^i^	180.0	O1^ii^—Na1—Mg1	84.21 (4)
O8—Mg1—O9	90.0	O6—Na1—Mg1	144.34 (6)
O8^i^—Mg1—O9	90.0	O5—Na1—Mg1	33.73 (3)
O8—Mg1—O9^i^	90.0	O6^iii^—Na1—Mg1	140.26 (5)
O8^i^—Mg1—O9^i^	90.0	O9^i^—Na1—Mg1	34.15 (3)
O9—Mg1—O9^i^	180.00 (8)	O1—Na1—Na1^iii^	95.68 (4)
O8—Mg1—O5^i^	90.0	O1^ii^—Na1—Na1^iii^	95.68 (4)
O8^i^—Mg1—O5^i^	90.0	O6—Na1—Na1^iii^	38.69 (5)
O9—Mg1—O5^i^	86.23 (6)	O5—Na1—Na1^iii^	143.24 (5)
O9^i^—Mg1—O5^i^	93.77 (6)	O6^iii^—Na1—Na1^iii^	36.70 (4)
O8—Mg1—O5	90.0	O9^i^—Na1—Na1^iii^	148.88 (5)
O8^i^—Mg1—O5	90.0	Mg1—Na1—Na1^iii^	176.97 (4)
O9—Mg1—O5	93.77 (6)	C5—O1—Na1	175.97 (11)
O9^i^—Mg1—O5	86.23 (6)	C4—C1—C4^iv^	125.01 (18)
O5^i^—Mg1—O5	180.0	C4—C1—H1	117.5
O8—Mg1—Na1^i^	90.0	C4^iv^—C1—H1	117.5
O8^i^—Mg1—Na1^i^	90.0	C6—O2—H3	115.2
O9—Mg1—Na1^i^	44.36 (4)	C3—C2—C3^iv^	125.01 (17)
O9^i^—Mg1—Na1^i^	135.64 (4)	C3—C2—H2	117.5
O5^i^—Mg1—Na1^i^	41.87 (4)	C3^iv^—C2—H2	117.5
O5—Mg1—Na1^i^	138.13 (4)	C5—O3—H3	114.6
O8—Mg1—Na1	90.0	C2—C3—C4	117.42 (12)
O8^i^—Mg1—Na1	90.0	C2—C3—C6	114.02 (12)
O9—Mg1—Na1	135.64 (4)	C4—C3—C6	128.56 (11)
O9^i^—Mg1—Na1	44.36 (4)	C1—C4—C3	117.57 (12)
O5^i^—Mg1—Na1	138.13 (4)	C1—C4—C5	113.34 (13)
O5—Mg1—Na1	41.87 (4)	C3—C4—C5	129.09 (11)
Na1^i^—Mg1—Na1	180.0	Mg1—O5—Na1	104.40 (6)
O1—Na1—O1^ii^	167.72 (7)	Mg1—O5—H5	114.8
O1—Na1—O6	95.89 (4)	Na1—O5—H5	112.0
O1^ii^—Na1—O6	95.89 (4)	O1—C5—O3	120.93 (13)
O1—Na1—O5	84.05 (4)	O1—C5—C4	119.43 (13)
O1^ii^—Na1—O5	84.05 (4)	O3—C5—C4	119.64 (12)
O6—Na1—O5	178.07 (7)	Na1—O6—Na1^iii^	104.61 (6)
O1—Na1—O6^iii^	93.16 (4)	Na1—O6—H6	116.0
O1^ii^—Na1—O6^iii^	93.16 (4)	Na1^iii^—O6—H6	103.9
O6—Na1—O6^iii^	75.39 (6)	O4—C6—O2	121.94 (13)
O5—Na1—O6^iii^	106.54 (6)	O4—C6—C3	118.58 (12)
O1—Na1—O9^i^	86.35 (4)	O2—C6—C3	119.48 (12)
O1^ii^—Na1—O9^i^	86.35 (4)	Mg1—O8—H8	127.2
O6—Na1—O9^i^	110.19 (6)	Mg1—O9—Na1^i^	101.49 (6)
O5—Na1—O9^i^	67.88 (5)	Mg1—O9—H9	117.8
O6^iii^—Na1—O9^i^	174.41 (6)	Na1^i^—O9—H9	109.5
O1—Na1—Mg1	84.21 (4)		

Symmetry codes: (i) −*x*, −*y*, −*z*; (ii) *x*, −*y*, *z*; (iii) −*x*+1, −*y*, −*z*+1; (iv) −*x*+1, *y*, −*z*+1.

### Hydrogen-bond geometry (Å, º)

**Table:**

*D*—H···*A*	*D*—H	H···*A*	*D*···*A*	*D*—H···*A*
O3—H3···O2	1.06	1.35	2.3827 (17)	163
O5—H5···O7	0.90	1.83	2.7313 (14)	173
O6—H6···O4^v^	0.90	1.97	2.8519 (15)	167
O7—H7···O2^vi^	0.86	1.91	2.7699 (14)	173
O8—H8···O3^vii^	0.85	1.94	2.7779 (14)	172
O9—H9···O4^vi^	0.85	1.91	2.7559 (14)	171

Symmetry codes: (v) −*x*+1/2, *y*−1/2, −*z*+1; (vi) −*x*+1/2, −*y*+1/2, −*z*; (vii) −*x*, *y*, −*z*.
